# Supplementary material for: Early statin use is associated with improved survival and cardiovascular outcomes in patients with atrial fibrillation and recent ischaemic stroke: A propensity-matched analysis of a global federated health database
Source: Eur Stroke J. 2024 Sep 10;10(1):116–27. doi: 10.1177/23969873241274213 (PMC11558656; doi:10.1177/23969873241274213)
Supplement: sj-docx-1-eso-10.1177_23969873241274213 – Supplemental material for Early statin use is associated with improved survival and cardiovascular outcomes in patients with atrial fibrillation and recent ischaemic stroke: A propensity-matched analysis of a global federated health database [file sj-docx-1-eso-10.1177_23969873241274213.docx]

**Early statin use is associated with improved survival and cardiovascular outcomes in patients with atrial fibrillation and recent ischaemic stroke.**

**A propensity-matched analysis of a global federated health database**

Sylvia E. Choi, MBBS^1,2^, Tommaso Bucci, MD, PhD^1,2,3^, Jia-yi Huang, MD^4,5^,

Kai-Hang YIU, MD, PhD^4,5^, Christopher T. W. Tsang, BSc^5^, Kui Kai Lau^6,7^, Andrew Hill, MBChB^1,8^, Greg Irving, PhD^1,9^, Gregory Y. H. Lip, MD^1,10*^, Azmil H. Abdul-Rahim, MD^1,2,8*†^

1) Liverpool Centre for Cardiovascular Science at University of Liverpool, Liverpool John Moores University and Liverpool Heart & Chest Hospital, Liverpool, UK.

2) Department of Cardiovascular and Metabolic Medicine, Institute of Life Course and Medical Sciences, University of Liverpool, Liverpool, UK.

3) Department of Clinical Internal, Anesthesiologic and Cardiovascular Sciences, Sapienza University of Rome, Rome, Italy..

4) Cardiology Division, Department of Medicine, The University of Hong Kong Shen Zhen Hospital, Shen Zhen, China.

5) Cardiology Division, Department of Medicine, Queen Mary Hospital, The University of Hong Kong, Hong Kong, China.

6) Division of Neurology, Department of Medicine, The University of Hong Kong, Hong Kong, China.

7) State Key Laboratory of Brain and Cognitive Sciences, The University of Hong Kong, Hong Kong, China.

8) Stroke Division, Department of Medicine for Older People, Whiston Hospital, Mersey and West Lancashire Teaching Hospitals NHS Trust, Prescot, UK.

9) Health Research Institute, Edge Hill University Faculty of Health and Social Care, Ormskirk, UK.

10) Danish Center for Health Services Research, Department of Clinical Medicine, Aalborg University, Denmark.

^*^ Joint senior authors
^†^ Author for correspondence: [Azmil.Abdul-Rahim@liverpool.ac.uk](mailto:Azmil.Abdul-Rahim@liverpool.ac.uk)

Address: Liverpool Centre for Cardiovascular Science, William Henry Duncan Building, 6 West Derby Street, Liverpool, L7 8TX, United Kingdom

**Supplementary Materials**

**Table S1**. STROBE Statement—checklist of items that should be included in reports of observational studies

|  | **Item No** | **Recommendation** | **Page  No** |
| --- | --- | --- | --- |
| **Title and abstract** | 1 | (*a*) Indicate the study’s design with a commonly used term in the title or the abstract | Page 4; abstract |
|  |  | (*b*) Provide in the abstract an informative and balanced summary of what was done and what was found | Page 4; abstract |
| **Introduction** | | | |
| Background/rationale | 2 | Explain the scientific background and rationale for the investigation being reported | Page 8; introduction |
| Objectives | 3 | State specific objectives, including any prespecified hypotheses | Page 9; end of introduction |
| **Methods** | | | |
| Study design | 4 | Present key elements of study design early in the paper | Page 9-13; methods |
| Setting | 5 | Describe the setting, locations, and relevant dates, including periods of recruitment, exposure, follow-up, and data collection | Page 9-13; methods |
| Participants | 6 | (*a*) Give the eligibility criteria, and the sources and methods of selection of participants. Describe methods of follow-up | Page 9-13; methods |
|  |  | (*b*) For matched studies, give matching criteria and number of exposed and unexposed | Page 9-13; methods |
| Variables | 7 | Clearly define all outcomes, exposures, predictors, potential confounders, and effect modifiers. Give diagnostic criteria, if applicable | Page 9-13; methods |
| Data sources/ measurement | 8* | For each variable of interest, give sources of data and details of methods of assessment (measurement). Describe comparability of assessment methods if there is more than one group | Page 9-13; methods + *Tables S3-4 and Table S1* |
| Bias | 9 | Describe any efforts to address potential sources of bias | Page 9-13; methods |
| Study size | 10 | Explain how the study size was arrived at | Page 9-13; methods |
| Quantitative variables | 11 | Explain how quantitative variables were handled in the analyses. If applicable, describe which groupings were chosen and why | Page 9-13; methods |
| Statistical methods | 12 | (*a*) Describe all statistical methods, including those used to control for confounding | Page 9-13; methods |
|  |  | (*b*) Describe any methods used to examine subgroups and interactions | Page 9-13; methods |
|  |  | (*c*) Explain how missing data were addressed | N/A |
|  |  | (*d*) If applicable, explain how loss to follow-up was addressed | N/A |
|  |  | (*e*) Describe any sensitivity analyses | Page 9-13; methods |
| **Results** |  |  |  |
| Participants | 13* | (a) Report numbers of individuals at each stage of study—eg numbers potentially eligible, examined for eligibility, confirmed eligible, included in the study, completing follow-up, and analysed | Page 14; results |
|  |  | (b) Give reasons for non-participation at each stage | N/A |
|  |  | (c) Consider use of a flow diagram | N/A |
| Descriptive data | 14* | (a) Give characteristics of study participants (eg demographic, clinical, social) and information on exposures and potential confounders | Page 14; results + *Tables S3-4* |
|  |  | (b) Indicate number of participants with missing data for each variable of interest | N/A |
|  |  | (c) Summarise follow-up time (eg, average and total amount) | Page 14; results |
| Outcome data | 15* | Report numbers of outcome events or summary measures over time | Page 14-19; results + *Figures 1-2* |
| Main results | 16 | (*a*) Give unadjusted estimates and, if applicable, confounder-adjusted estimates and their precision (eg, 95% confidence interval). Make clear which confounders were adjusted for and why they were included | Page 14-19; results + *Figures 1-2* |
|  |  | (*b*) Report category boundaries when continuous variables were categorized | N/A |
|  |  | (*c*) If relevant, consider translating estimates of relative risk into absolute risk for a meaningful time period | Page 14-19; results + *Figures 1-2* |
| Other analyses | 17 | Report other analyses done—eg analyses of subgroups and interactions, and sensitivity analyses | Page 14-19; results + *Figure 2* |
| **Discussion** | | | |
| Key results | 18 | Summarise key results with reference to study objectives | Page 19 |
| Limitations | 19 | Discuss limitations of the study, taking into account sources of potential bias or imprecision. Discuss both direction and magnitude of any potential bias | Page 25-27; strengths and limitations |
| Interpretation | 20 | Give a cautious overall interpretation of results considering objectives, limitations, multiplicity of analyses, results from similar studies, and other relevant evidence | Page 27; conclusions |
| Generalisability | 21 | Discuss the generalisability (external validity) of the study results | Page 19-25 |
| **Other information** | | | |
| Funding | 22 | Give the source of funding and the role of the funders for the present study and, if applicable, for the original study on which the present article is based | Page 28 |

*Give information separately for exposed and unexposed groups.

**Note:** An Explanation and Elaboration article discusses each checklist item and gives methodological background and published examples of transparent reporting. The STROBE checklist is best used in conjunction with this article (freely available on the Web sites of PLoS Medicine at http://www.plosmedicine.org/, Annals of Internal Medicine at http://www.annals.org/, and Epidemiology at http://www.epidem.com/). Information on the STROBE Initiative is available at www.strobe-statement.org.

**Table S2**. ICD-10-CM codes and other diagnostic, procedural and medication codes (indicated by the TriNetX website) used in the cohort definitions, outcomes definitions and covariates list

| **Cohort definitions** | **ICD-10-CM and other diagnostic codes** |
| --- | --- |
| Atrial fibrillation or flutter | I48 [Atrial fibrillation and flutter] |
| Ischaemic stroke | I63 [Cerebral infarction] |
| Statins | C10AA [HMG CoA reductase inhibitors] |
| Persistent and chronic AF | Any of the following:   - I48.1 [Persistent atrial fibrillation] - I48.2 [Chronic atrial fibrillation] |
| Paroxysmal AF | I48.0 [Paroxysmal atrial fibrillation] |
| LVO stroke | I63.5 [Cerebral infarction due to unspecified occlusion or stenosis of cerebral arteries] |
| Embolic stroke | I63.40 [Cerebral infarction due to embolism of unspecified cerebral artery] |
| Hypertensive diseases | Any of the following:   - I10 [Essential (primary) hypertension] - I11 [Hypertensive heart disease] - I12 [Hypertensive chronic kidney disease] - I13 [Hypertensive heart and chronic kidney disease] - I15 [Secondary hypertension] - I16 [Hypertensive crisis] |
| Ischaemic heart diseases | Any of the following:   - I20 [Angina pectoris] - I21 [Acute myocardial infarction] - I22 [Subsequent ST elevation (STEMI) and non-ST elevation (NSTEMI) myocardial infarction - I23 [Certain current complications following ST elevation (STEMI) and non-ST elevation (NSTEMI) myocardial infarction (within the 28 day period)] - I24 [Other acute ischemic heart diseases] - I25 [Chronic ischemic heart disease] |
| Type 2 diabetes mellitus | E11 [Type 2 diabetes mellitus] |
| Dyslipidaemia | E78 [Disorders of lipoprotein metabolism and other lipidemias] |
| IVT | Any of the following:   - ICD-10-CM PCS: 3E03317 [Introduction of Other Thrombolytic into Peripheral Vein, Percutaneous Approach] - ICD-10-CM PCS: 3E05317 [Introduction of Other Thrombolytic into Peripheral Artery, Percutaneous Approach] - ICD-10-CM: Z92.82 [Status post administration of tPA (rtPA) in a different facility within the last 24 hours prior to admission to current facility] - CPT:37195 [Thrombolysis, cerebral, by intravenous infusion] - SNOMED 23093400 [Thrombolysis of intracranial vessel] |
| EVT | Any of the following:   - ICD-10-CM PCS: 03CG3Z7 [Extirpation of Matter from Intracranial Artery using Stent Retriever, Percutaneous Approach] - ICD-10-CM PCS: 03CG3ZZ [Extirpation of Matter from Intracranial Artery, Percutaneous Approach] - ICD-10-CM PCS: 03CG4ZZ [Extirpation of Matter from Intracranial Artery, Percutaneous Endoscopic Approach] - ICD-10-CM PCS: 03CG3Z6 [Extirpation of Matter from Intracranial Artery, Bifurcation, Percutaneous Approach (deprecated 2020)] - ICD-10-CM PCS: 03CG3Z7 [Extirpation of Matter from Intracranial Artery using Stent Retriever, Percutaneous Approach] - ICD-10-CM PCS: 03CG3ZZ [Extirpation of Matter from Intracranial Artery, Percutaneous Approach] - ICD-10-CM PCS: 03CG4Z6 [Extirpation of Matter from Intracranial Artery, Bifurcation, Percutaneous Endoscopic Approach (deprecated 2020)] - ICD-10-CM PCS: 03CG4ZZ [Extirpation of Matter from Intracranial Artery, Percutaneous Endoscopic Approach] - ICD-10-CM PCS: 03CK3Z6 [Extirpation of Matter from Right Internal Carotid Artery, Bifurcation, Percutaneous Approach (deprecated 2020)] - ICD-10-CM PCS: 03CK3Z7 [Extirpation of Matter from Right Internal Carotid Artery using Stent Retriever, Percutaneous Approach] - ICD-10-CM PCS: 03CK3ZZ [Extirpation of Matter from Right Internal Carotid Artery, Percutaneous Approach] - ICD-10-CM PCS: 03CK4Z6 [Extirpation of Matter from Right Internal Carotid Artery, Bifurcation, Percutaneous Endoscopic Approach (deprecated 2020)] - ICD-10-CM PCS: 03CK4ZZ [Extirpation of Matter from Right Internal Carotid Artery, Percutaneous Endoscopic Approach] - ICD-10-CM PCS: 03CL3Z6 [Extirpation of Matter from Left Internal Carotid Artery, Bifurcation, Percutaneous Approach (deprecated 2020)] - ICD-10-CM PCS: 03CL3Z7 [Extirpation of Matter from Left Internal Carotid Artery using Stent Retriever, Percutaneous Approach] - ICD-10-CM PCS: 03CL3ZZ [Extirpation of Matter from Left Internal Carotid Artery, Percutaneous Approach] - ICD-10-CM PCS: 03CL4Z6 [Extirpation of Matter from Left Internal Carotid Artery, Bifurcation, Percutaneous Endoscopic Approach (deprecated 2020)] - ICD-10-CM PCS: 03CL4ZZ [Extirpation of Matter from Left Internal Carotid Artery, Percutaneous Endoscopic Approach] - ICD-10-CM PCS: 03CP3Z6 [Extirpation of Matter from Right Vertebral Artery, Bifurcation, Percutaneous Approach (deprecated 2020)] - ICD-10-CM PCS: 03CP3Z7 [Extirpation of Matter from Right Vertebral Artery using Stent Retriever, Percutaneous Approach] - ICD-10-CM PCS: 03CP3ZZ [Extirpation of Matter from Right Vertebral Artery, Percutaneous Approach] - ICD-10-CM PCS: 03CP4ZZ [Extirpation of Matter from Right Vertebral Artery, Percutaneous Endoscopic Approach] - ICD-10-CM PCS: 03CQ3Z6 [Extirpation of Matter from Left Vertebral Artery, Bifurcation, Percutaneous Approach (deprecated 2020)] - ICD-10-CM PCS: 03CQ3Z7 [Extirpation of Matter from Left Vertebral Artery using Stent Retriever, Percutaneous Approach] - ICD-10-CM PCS: 03CQ3ZZ [Extirpation of Matter from Left Vertebral Artery, Percutaneous Approach] - ICD-10-CM PCS: 03CQ4ZZ [Extirpation of Matter from Left Vertebral Artery, Percutaneous Endoscopic Approach] - SNOMED: 21710002 [Removal of thrombus from intracranial artery] - SNOMED: 439541007 [Percutaneous thrombectomy of cerebral artery using fluoroscopic guidance with contrast] - CPT:61645 [Percutaneous arterial transluminal mechanical thrombectomy and/or infusion for thrombolysis, intracranial, any method, including diagnostic angiography, fluoroscopic guidance, catheter placement, and intraprocedural pharmacological thrombolytic injection(s)] |
| **Outcomes definitions** | **ICD-10-CM and other diagnostic codes** |
| Ischaemic stroke | I63 [Cerebral infarction] |
| All-cause mortality | Deceased |
| Composite outcome | The composite of any of the following:   - I63 [Cerebral infarction] - G45 [Transient cerebral ischemic attacks and related syndromes] - I21 [Acute myocardial infarction] - Deceased |
| Transient ischaemic attack | G45 [Transient cerebral ischemic attacks and related syndromes] |
| Intracranial haemorrhage | Any of the following:   - I61 [Nontraumatic intracerebral hemorrhage] - I62 [Other and unspecified nontraumatic intracranial hemorrhage] |
| Acute myocardial infarction | I21 [Acute myocardial infarction] |
| Hospital readmission | 1013659 [Hospital Inpatient Services] |
| **Covariates list** | **ICD-10-CM and other diagnostic codes** |
| Hypertensive diseases | Any of the following:   - I10 [Essential (primary) hypertension] - I11 [Hypertensive heart disease] - I12 [Hypertensive chronic kidney disease] - I13 [Hypertensive heart and chronic kidney disease] - I15 [Secondary hypertension] - I16 [Hypertensive crisis] |
| Ischaemic heart diseases | Any of the following:   - I20 [Angina pectoris] - I21 [Acute myocardial infarction] - I22 [Subsequent ST elevation (STEMI) and non-ST elevation (NSTEMI) myocardial infarction - I23 [Certain current complications following ST elevation (STEMI) and non-ST elevation (NSTEMI) myocardial infarction (within the 28 day period)] - I24 [Other acute ischemic heart diseases] - I25 [Chronic ischemic heart disease] |
| Heart failure | I50 [Heart failure] |
| Dyslipidaemia | E78 [Disorders of lipoprotein metabolism and other lipidemias] |
| Atrial fibrillation/flutter | I48 [Atrial fibrillation and flutter] |
| Peripheral vascular disease | I73 [Other peripheral vascular diseases] |
| Pulmonary heart disease | Pulmonary heart disease and diseases of pulmonary circulation, being any of:   - I26 [Pulmonary embolism] - I27 [Other pulmonary heart diseases] - I28 [Other diseases of pulmonary vessels] |
| Type 2 diabetes mellitus | E11 [Type 2 diabetes mellitus] |
| Chronic kidney disease | N18 [Chronic kidney disease (CKD)] |
| Neoplasms | C00-D49  Any of the following:   - C00-C14 [Malignant neoplasms of lip, oral cavity and pharynx] - C15-C26 [Malignant neoplasms of digestive organs] - C30-C39 [Malignant neoplasms of respiratory and intrathoracic organs] - C40-C41 [Malignant neoplasms of bone and articular cartilage] - C43-44 [Melanoma and other malignant neoplasms of skin] - C45-C49 [Malignant neoplasms of mesothelial and soft tissue] - C50 [Malignant neoplasms of breast] - C51-C58 [Malignant neoplasms of female genital organs] - C60-C63 [Malignant neoplasms of male genital organs] - C64-C68 [Malignant neoplasms of urinary tract] - C69-C72 [Malignant neoplasms of eye, brain and other parts of central nervous system] - C73-C75 [Malignant neoplasms of thyroid and other endocrine glands] - C76-C80 [Malignant neoplasms of ill-defined, other secondary and unspecified sites] - C7A [Malignant neuroendocrine tumors] - C7B [Secondary neuroendocrine tumors] - C81-C96 [Malignant neoplasms of lymphoid, hematopoietic and related tissue] - D00-D09 [In situ neoplasms] - D10-D36 [Benign neoplasms, except benign neuroendocrine tumors] - D37-D48 [Neoplasms of uncertain behavior, polycythemia vera and myelodysplastic syndromes] - D3A [Benign neuroendocrine tumors] - D49 [Neoplasms of unspecified behavior] |
| Overweight and obesity | E66 [Overweight and obesity] |
| Obstructive sleep apnoea | G47.33 [Obstructive sleep apnea (adult) (pediatric)] |
|  | *Medications* |
| Beta-blockers | CV100 [Beta blockers/related] |
| ACE inhibitors | CV800 [ACE inhibitors] |
| Angiotensin II Inhibitors | CV805 [Angiotensin II Inhibitors] |
| Calcium channel blockers | CV200 [Calcium channel blockers] |
| Diuretics | CV700 [Diuretics] |
| Antiplatelet therapy | BL117 [Platelet aggregation inhibitors] |
| Anticoagulation | BL110 [Anticoagulants] |
| Insulin | HS501 [Insulin] |
| Oral hypoglycaemic agents | HS502 [Oral hypoglycaemic agents, oral] |
| ACE = angiotensin-converting enzyme; AF = atrial fibrillation; DC = direct current; EP = electrophysiological; EVT = endovascular thrombectomy; ICD = implantable cardioverter-defibrillator; ICD-10-CM = International Classification of Diseases, Tenth Revision, Clinical Modification; IVT = intravenous thrombolysis | |

**Table S3**. Baseline characteristics of individuals in the early statin and no early statin groups *before* and *after* propensity score matching*

|  | **Before propensity score matching** | | | **After propensity score matching** | | |
| --- | --- | --- | --- | --- | --- | --- |
|  | Early statin (n=7,500) | No early statin (n=13,402) | SMD | Early statin (n=5,591) | No early statin (n=5,591) | SMD |
| *Demographics* |  |  |  |  |  |  |
| Age (years), mean ± sd | 73.8 ± 11.1 | 73.5 ± 12.2 | 0.027 | 73.7 ± 11.1 | 73.7 ± 11.9 | 0.001 |
| Female, *n (%)* | 3,494 (46.6) | 6,461 (48.3) | 0.034 | 2,652 (47.4) | 2,625 (47.0) | 0.010 |
| Race, *n (%)* |  |  |  |  |  |  |
| White ethnicity | 5,232 (69.8) | 8,654 (64.7) | 0.109 | 3,830 (68.5) | 3,852 (68.9) | 0.008 |
| Black or African American | 803 (10.7) | 1,592 (11.9) | 0.038 | 603 (10.8) | 577 (10.3) | 0.015 |
| Hispanic or Latino | 248 (3.3) | 471 (3.5) | 0.012 | 198 (3.5) | 208 (3.7) | 0.010 |
| Asian | 231 (3.1) | 397 (3.0) | 0.007 | 175 (3.1) | 179 (3.2) | 0.004 |
| *Comorbidities, n (%)* |  |  |  |  |  |  |
| Hypertensive diseases | 3,693 (49.2) | 9.424 (70.4) | 0.442 | 3,410 (61.0) | 3,460 (61.9) | 0.018 |
| Ischaemic heart diseases | 1,797 (24.0) | 4,881 (36.5) | 0.275 | 1,673 (29.9) | 1,718 (30.7) | 0.018 |
| Heart failure | 1,210 (16.1) | 4,142 (30.9) | 0.355 | 1,149 (20.6) | 1,193 (21.3) | 0.019 |
| Dyslipidaemia | 2,420 (32.3) | 5,468 (40.9) | 0.179 | 2,199 (39.3) | 2,216 (39.6) | 0.006 |
| Atrial fibrillation/flutter | 4,219 (56.3) | 11,298 (84.4) | 0.648 | 3,966 (70.9) | 4,004 (71.6) | 0.015 |
| Peripheral vascular disease | 269 (3.6) | 799 (6.0) | 0.112 | 254 (4.5) | 234 (4.2) | 0.018 |
| Pulmonary heart disease | 302 (4.0) | 1,053 (7.9) | 0.163 | 282 (5.0) | 289 (5.2) | 0.006 |
| Type 2 diabetes mellitus | 1,597 (21.3) | 4,305 (32.2) | 0.248 | 1,470 (26.3) | 1,539 (27.5) | 0.028 |
| Chronic kidney disease | 526 (7.0) | 2,423 (18.1) | 0.339 | 516 (9.2) | 570 (10.2) | 0.033 |
| Neoplasms | 355 (4.7) | 1,494 (11.2) | 0.239 | 348 (6.2) | 345 (6.2) | 0.002 |
| Overweight and obesity | 434 (5.8) | 1,367 (10.2) | 0.164 | 412 (7.4) | 437 (7.8) | 0.017 |
| Obstructive sleep apnoea | 243 (3.2) | 943 (7.0) | 0.173 | 237 (4.2) | 237 (4.2) | <0.001 |
| *Medications, n (%)* |  |  |  |  |  |  |
| Beta-blockers | 4,706 (62.7) | 6,057 (45.3) | 0.356 | 3,346 (59.8) | 3,390 (60.6) | 0.016 |
| ACE Inhibitors | 2,976 (39.7) | 4,191 (31.3) | 0.175 | 2,207 (39.5) | 2,192 (39.2) | 0.005 |
| Angiotensin II Inhibitors | 1,332 (17.8) | 2,314 (17.3) | 0.012 | 1,043 (18.7) | 1,107 (19.8) | 0.029 |
| Calcium channel blockers | 2,198 (29.3) | 3,077 (23.0) | 0.144 | 1,608 (28.8) | 1,615 (28.9) | 0.003 |
| Diuretics | 2,456 (32.7) | 4,013 (30.0) | 0.060 | 1,852 (33.1) | 1,910 (34.2) | 0.022 |
| Antiplatelet therapy | 4,292 (57.2) | 4,999 (37.4) | 0.406 | 2,886 (51.6) | 2,955 (52.9) | 0.025 |
| Anticoagulation | 4,553 (60.7) | 5,616 (42.0) | 0.382 | 3,118 (55.8) | 3,167 (56.6) | 0.018 |
| Insulin | 2,222 (29.6) | 2,892 (21.6) | 0.184 | 1,539 (27.5) | 1,578 (28.2) | 0.016 |
| Oral hypoglycaemic agents | 786 (10.5) | 1,062 (7.9) | 0.088 | 575 (10.3) | 602 (10.8) | 0.016 |
|  |  |  |  |  |  |  |
| ACE = angiotensin-converting enzyme; DC = direct current; EP = electrophysiological; ICD = implantable cardioverter-defibrillator; sd = standard deviation; SMD = standardised mean difference.  *A greedy nearest neighbour matching model was performed with a caliper of 0.1 pooled standard deviations using logistic regression. Any baseline characteristic with a SMD between cohorts lower than 0.1 is considered well matched | | | | | | |

**Table S4**. Baseline characteristics of individuals aged 75 years and over in the early statin and no early statin groups *before* and *after* propensity score matching*

|  | **Before propensity score matching** | | | **After propensity score matching** | | |
| --- | --- | --- | --- | --- | --- | --- |
|  | Early statin (n=5,762) | No early statin (n=10,078) | SMD | Early statin (n=4,313) | No early statin (n=4,313) | SMD |
| *Demographics* |  |  |  |  |  |  |
| Age (years), mean ± sd | 78.5 ± 7.1 | 79.0 ± 7.3 | 0.063 | 78.5 ± 7.1 | 78.5 ± 7.3 | 0.002 |
| Female, *n (%)* | 2,953 (51.2) | 5,306 (52.7) | 0.030 | 2,237 (51.9) | 2,211 (51.3) | 0.012 |
| Race, *n (%)* |  |  |  |  |  |  |
| White ethnicity | 4,157 (72.1) | 6,864 (68.2) | 0.086 | 3,081 (71.4) | 3,073 (71.2) | 0.004 |
| Black or African American | 474 (8.2) | 862 (8.6) | 0.012 | 351 (8.1) | 360 (8.3) | 0.008 |
| Hispanic or Latino | 163 (2.8) | 301 (3.0) | 0.010 | 131 (3.0) | 146 (3.4) | 0.020 |
| Asian | 181 (3.1) | 300 (3.0) | 0.009 | 135 (3.1) | 130 (3.0) | 0.007 |
| *Comorbidities, n (%)* |  |  |  |  |  |  |
| Hypertensive diseases | 2,837 (49.2) | 7,136 (70.9) | 0.454 | 2,642 (61.3) | 2,665 (61.8) | 0.011 |
| Ischaemic heart diseases | 1,397 (24.2) | 3,636 (36.1) | 0.261 | 1,304 (30.2) | 1,369 (31.7) | 0.033 |
| Heart failure | 885 (15.4) | 2,925 (29.1) | 0.334 | 849 (19.7) | 880 (20.4) | 0.018 |
| Dyslipidaemia | 1,880 (32.6) | 4,146 (41.2) | 0.179 | 1,720 (39.9) | 1,759 (40.8) | 0.018 |
| Atrial fibrillation/flutter | 3,264 (56.6) | 8,540 (84.9) | 0.653 | 3,091 (71.7) | 3,105 (72.0) | 0.007 |
| Peripheral vascular disease | 221 (3.8) | 608 (6.0) | 0.102 | 212 (4.9) | 217 (5.0) | 0.005 |
| Pulmonary heart disease | 235 (4.1) | 730 (7.3) | 0.138 | 218 (5.1) | 219 (5.1) | 0.001 |
| Type 2 diabetes mellitus | 1,136 (19.7) | 3,060 (30.4) | 0.249 | 1,060 (24.6) | 1,114 (25.8) | 0.029 |
| Chronic kidney disease | 407 (7.1) | 1,778 (17.7) | 0.327 | 397 (9.2) | 427 (9.9) | 0.024 |
| Neoplasms | 307 (5.3) | 1,174 (11.7) | 0.229 | 305 (7.1) | 329 (7.6) | 0.021 |
| Overweight and obesity | 236 (4.1) | 773 (7.7) | 0.153 | 228 (5.3) | 245 (5.7) | 0.017 |
| Obstructive sleep apnoea | 133 (2.3) | 536 (5.3) | 0.158 | 131 (3.0) | 135 (3.1) | 0.005 |
| *Medications, n (%)* |  |  |  |  |  |  |
| Beta-blockers | 3,617 (62.8) | 4,542 (45.1) | 0.359 | 2,590 (60.1) | 2,645 (61.3) | 0.026 |
| ACE Inhibitors | 2,264 (39.3) | 3,123 (31.0) | 0.173 | 1,674 (38.8) | 1,698 (39.4) | 0.011 |
| Angiotensin II Inhibitors | 1,124 (19.5) | 1,860 (18.5) | 0.026 | 897 (20.8) | 925 (21.4) | 0.016 |
| Calcium channel blockers | 1,695 (29.4) | 2,339 (23.2) | 0.140 | 1,253 (29.1) | 1,307 (30.3) | 0.027 |
| Diuretics | 1,883 (32.7) | 2,960 (29.4) | 0.070 | 1,427 (33.1) | 1,455 (33.7) | 0.014 |
| Antiplatelet therapy | 3,281 (56.9) | 3,810 (37.9) | 0.389 | 2,235 (51.8) | 2,293 (53.2) | 0.027 |
| Anticoagulation | 3,430 (59.5) | 4,134 (41.1) | 0.375 | 2,378 (55.1) | 2,412 (55.9) | 0.016 |
| Insulin | 1,628 (28.3) | 1,989 (19.8) | 0.200 | 1,113 (25.8) | 1,133 (26.3) | 0.011 |
| Oral hypoglycaemic agents | 616 (10.7) | 817 (8.1) | 0.088 | 467 (10.8) | 483 (11.2) | 0.012 |
|  |  |  |  |  |  |  |
| ACE = angiotensin-converting enzyme; DC = direct current; EP = electrophysiological; ICD = implantable cardioverter-defibrillator; sd = standard deviation; SMD = standardised mean difference.  *A greedy nearest neighbour matching model was performed with a caliper of 0.1 pooled standard deviations using logistic regression. Any baseline characteristic with a SMD between cohorts lower than 0.1 is considered well matched | | | | | | |

| Figure S1: Sensitivity and subgroup analyses using Cox regression |
| --- |
| 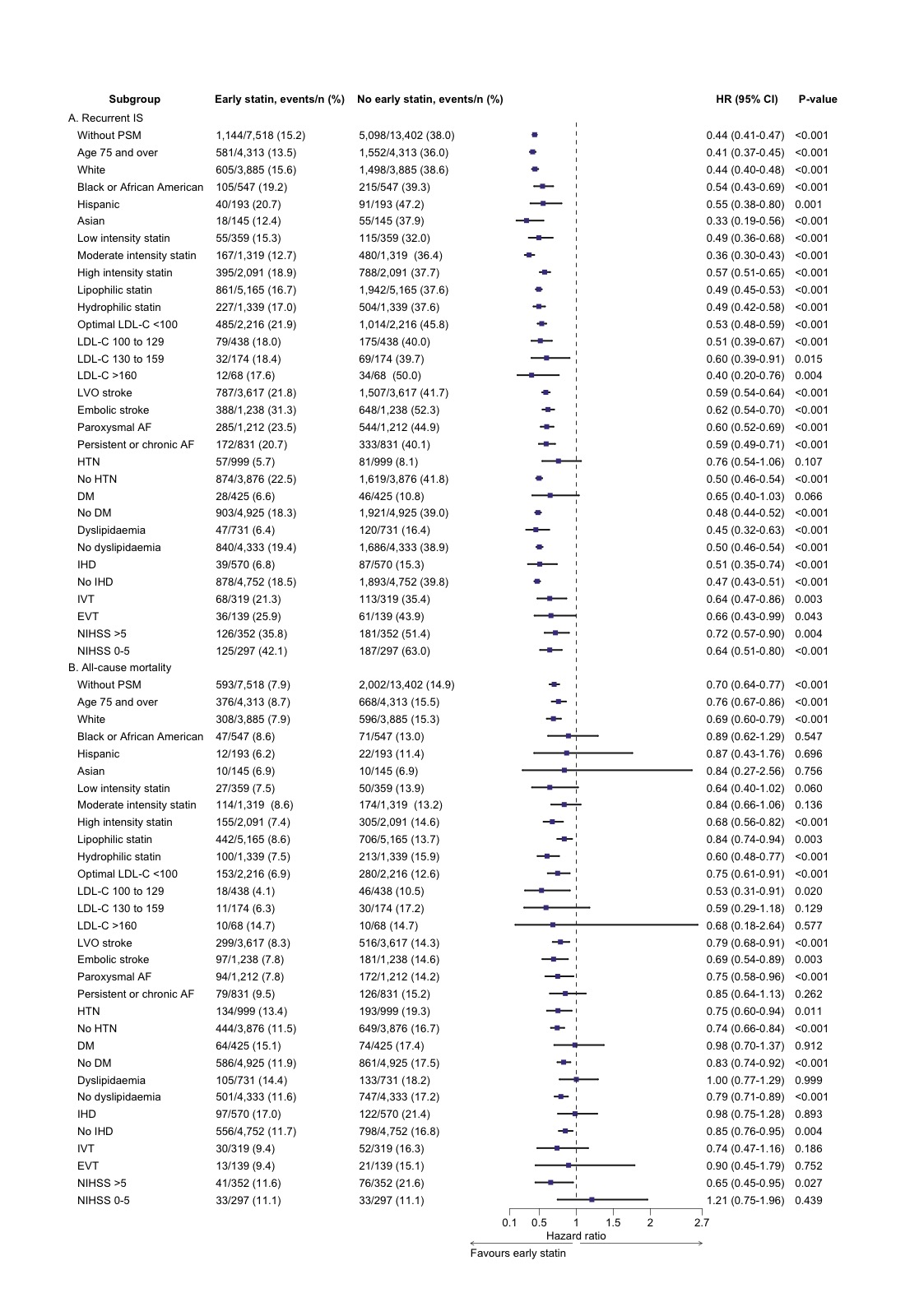  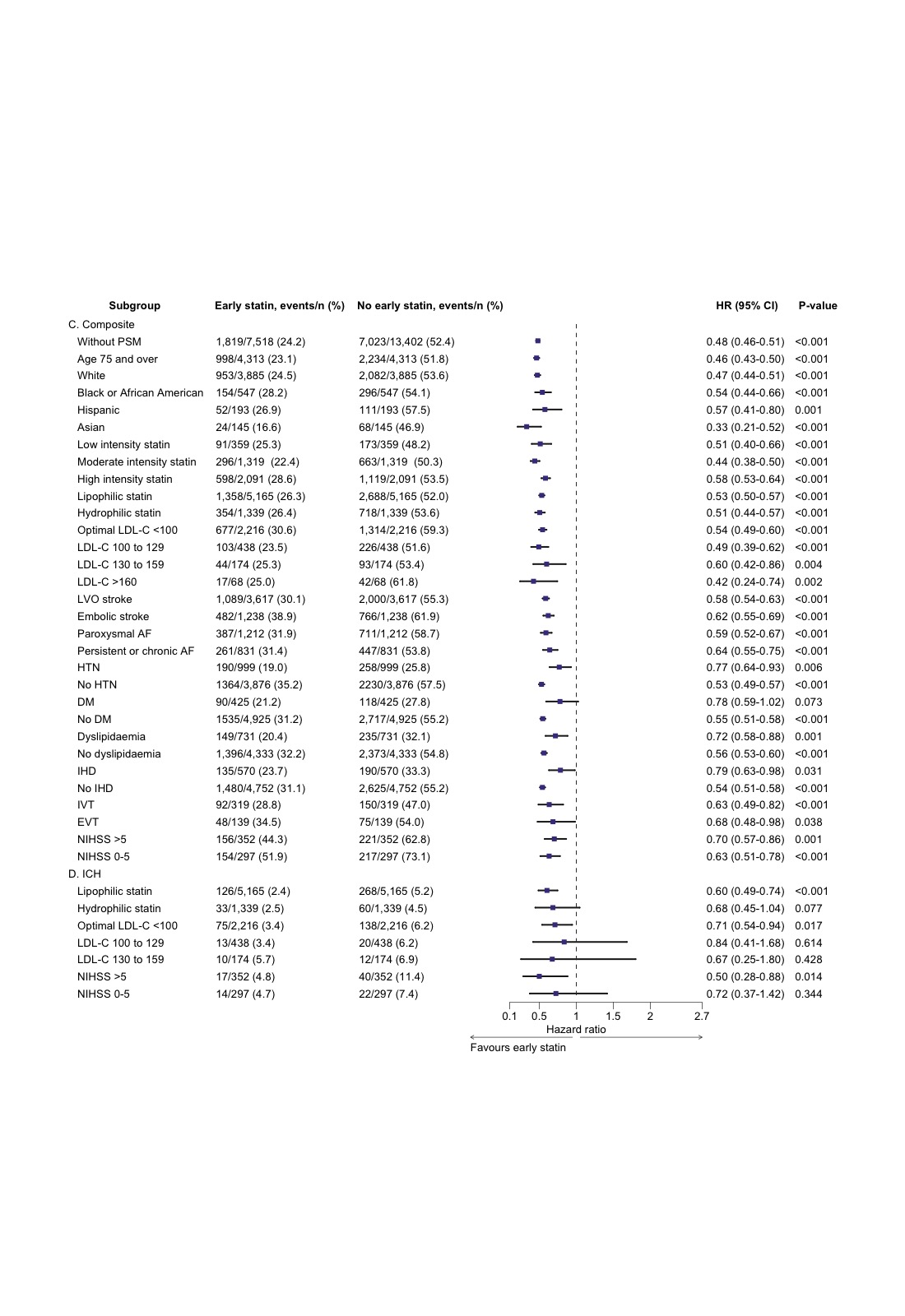 |
| Cox Regression is applied after propensity score matching (except in the sensitivity analysis of groups without propensity score matching). AF = atrial fibrillation; DM = type 2 diabetes mellitus; EVT = endovascular thrombectomy; HR = Hazard Ratio; HTN = hypertension; ICH = intracranial haemorrhage; IHD = ischaemic heart disease; IS = ischaemic stroke; IVT = intravenous thrombolysis; LDL-C = low density lipoprotein cholesterol (in mg/dL); LVO = large vessel occlusion; NIHSS = National Institutes of Health Stroke Scale score; PSM = propensity score matching; 95%CI = 95% Confidence Interval |
